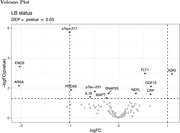# Plasma proteome profile of Lewy body pathology‐positive individuals

**DOI:** 10.1002/alz.095217

**Published:** 2025-01-09

**Authors:** Bárbara Fernandes Gomes, Andrea L. Benedet, Alessandro Padovani, Chiara Tolassi, Gianluigi Zanusso, Matilde Bongianni, Fabio Moda, Kaj Blennow, Henrik Zetterberg, Andrea Pilotto, Pedro Rosa‐Neto, Nicholas J. Ashton

**Affiliations:** ^1^ Department of Psychiatry and Neurochemistry, Institute of Neuroscience and Physiology, The Sahlgrenska Academy, University of Gothenburg, Mölndal, Gothenburg Sweden; ^2^ Neurology Unit, Department of Clinical and Experimental Sciences, University of Brescia, Brescia Italy; ^3^ University of Verona, Verona Italy; ^4^ Fondazione IRCSS Istituto Neurologico Carlo Besta, Milan Italy; ^5^ Translational Neuroimaging Laboratory, The McGill University Research Centre for Studies in Aging, Montréal, QC Canada

## Abstract

**Background:**

Lewy bodies (LB), the main hallmark of Parkinson’s disease (PD), are a frequent co‐pathology in Alzheimer’s disease (AD) and dementia with Lewy bodies (DLB). The varying extents of LB pathology in these disorders can influence disease progression and severity. Consequently, understanding LB impact on the proteomic profile of these diseases is crucial, potentially leading to identifyng novel blood biomarkers related to this pathology which are urgently needed. The aim of this project was to assess novel NULISAseq™ α‐synuclein (αSyn) biomarkers in blood. Considering the prevalence of monomeric αSyn in blood, we investigated whether other plasma proteins identified via NULISAseq™ or protein models could serve as surrogates for LB pathology determined by real‐time quake‐inducing conversion seed amplification assay (SAA).

**Method:**

The proteomic profile of two independent cohorts was assessed using the NULISAseq™ CNS Disease Panel (Alamar Biosciences). A pilot cohort included amyloid‐β (Aβ)‐positive and SAA‐negative AD patients (n = 30) and Aβ‐negative SAA‐positive patients (DLB, n = 22; PD, n = 2). The TRIAD cohort included young (n = 32), cognitively unimpaired Aβ‐positive and Aβ‐negative (CU+, n = 111; CU‐, n = 26) individuals, Aβ‐positive and Aβ‐negative mild cognitive impairment (MCI+, n = 40; MCI‐, n = 39), as well as AD dementia (ADD, n = 38) and non‐AD (n = 27) patients. LB pathology was confirmed in both cohorts by the same SAA method.

**Result:**

In the pilot cohort, no differences in plasma αSyn (SNCA, pSyn129, Oligo‐Syn) levels were observed. However, plasma phosphorylated tau‐217 (pTau‐217, logFoldChange (FC) = ‐1.00, *p.adj*<0.001), enolase‐2 (ENO2, logFC = ‐2.03, *p.adj* = 0.02), Fms Related Receptor Tyrosine Kinase 1 (FLT1, logFC = 0.54, *p.adj* = 0.04), and amyloid‐β42 (Aβ42, logFC = 1.09, *p.adj* = 0.04) were differentially expressed in SAA‐negative vs SAA‐positive patients. Furthermore, the ratio of pTau‐217/FLT1 exhibited the best separation between the SAA‐positive and SAA‐negative groups (AUC *=* 0.90, 95% CI 0.82‐0.98). The TRIAD cohort was used to validate these results, from which data is to be shown.

**Conclusion:**

The proteomic profile of LB‐positive is distinct from LB‐negative individuals, with four biomarkers differentially expressed in plasma. We also determined that a combination of these biomarkers may be useful to discriminate SAA‐positive from SAA‐negative individuals. Plasma αSyn levels did not reflect CSF SAA outcome, possibly due to peripheral αSyn expression not reflecting brain pathology.